# Modelling the interaction of steroid receptors with endocrine disrupting chemicals

**DOI:** 10.1186/1471-2105-6-S4-S10

**Published:** 2005-12-01

**Authors:** Pasqualina D'Ursi, Erika Salvi, Paola Fossa, Luciano Milanesi, Ermanna Rovida

**Affiliations:** 1 Department of Biomedical Sciences and Technologies, University of Milan, Segrate, Milan, 20090, Italy; 2 Institute for Biomedical Technologies, National Research Council, Segrate, Milan, 20090, Italy; 3 Department of Pharmaceutical Sciences, University of Genova, Genova 16132, Italy

## Abstract

**Background:**

The organic polychlorinated compounds like dichlorodiphenyltrichloroethane with its metabolites and polychlorinated biphenyls are a class of highly persistent environmental contaminants. They have been recognized to have detrimental health effects both on wildlife and humans acting as endocrine disrupters due to their ability of mimicking the action of the steroid hormones, and thus interfering with hormone response. There are several experimental evidences that they bind and activate human steroid receptors. However, despite the growing concern about the toxicological activity of endocrine disrupters, molecular data of the interaction of these compounds with biological targets are still lacking.

**Results:**

We have used a flexible docking approach to characterize the molecular interaction of seven endocrine disrupting chemicals with estrogen, progesterone and androgen receptors in the ligand-binding domain. All ligands docked in the buried hydrophobic cavity corresponding to the hormone steroid pocket. The interaction was characterized by multiple hydrophobic contacts involving a different number of residues facing the binding pocket, depending on ligands orientation. The EDC ligands did not display a unique binding mode, probably due to their lipophilicity and flexibility, which conferred them a great adaptability into the hydrophobic and large binding pocket of steroid receptors.

**Conclusion:**

Our results are in agreement with toxicological data on binding and allow to describe a pattern of interactions for a group of ECD to steroid receptors suggesting the requirement of a hydrophobic cavity to accommodate these chlorine carrying compounds. Although the affinity is lower than for hormones, their action can be brought about by a possible synergistic effect.

## Background

The organic polychlorinated compounds like dichlorodiphenyltrichloroethane (DDT) with its metabolites (DDx) and polychlorinated biphenyls (PCBs) belongs to a large family of highly persistent environmental pollutants that are toxic for the endocrine function. They accumulate in body tissues and fluids of exposed organisms, including humans, and, because of their lipophilic nature, are found at higher levels in adipose tissue, from which are released upon weight loss [1]. These compounds have been recognized to have detrimental health effects both on wildlife and humans. Acting as endocrine disrupters (EDC), they have been associated with several reproductive disorders in animals and humans [2] and have become an important environmental concern. The alarm is also related to the possible carcinogenicity of these compounds for hormone related tumours. Endocrine disrupting effect is obtained by mimicking the action of the steroid hormones, with which they share chemical characteristics as the hydrophobicity and the presence of an aromatic ring, and thus by interfering with the hormone response. Depending on the receptor targeted, they can exert agonistic or antagonistic effects. Moreover, synthesis and metabolism of natural hormones and levels of hormone receptors can be altered by EDC [3]. Experimental data "in vitro" show that DDT, PCB and metabolites compete with estradiol for the binding with α and β estrogen receptors with a relative affinity 1000–10000 times lower [4]. Evidence of binding has been also reported for androgen and progesterone receptors [5].

Despite the growing concern and the large amount of literature on the toxicological activity of EDC, molecular data of the interaction of these compounds with biological targets are still lacking.

With the aim to get insights, at molecular level, into the binding mode of selected EDC to target receptors, we have realized a set of computer-generated 3D models of human estrogen, androgen and progesterone receptors complexes with DDT and its metabolites DDE and DDD, and with the PCB hydroxylated derivative (PCB-OH). The site of interaction and the ligand conformation were predicted by the use of molecular docking techniques. The results of this computational study are here reported.

## Results and discussion

### Description of steroid receptors binding pocket

The crystal structures of ligand binding domains (LDBs) of the steroid receptors in complex with the corresponding natural hormones were used in this study: human alpha estrogen receptor (hERα) in complex with estradiol [6], human progesterone receptor (hPR) in complex with progesterone [7] and rat androgen receptor (rAR) in complex with dihydroxytestosterone [8].

Although several other X-ray complexes were available in PDB, we decided to restrict the choice to the natural steroid ligands to use target proteins under physiological conditions. It is in fact known that steroid receptors undergo to conformational changes after ligand binding and that different ligands are responsible for different conformations. For example it has been reported that the binding of antagonists (i.e. raloxifene) induce a conformational change in estrogen receptor ligand region through the displacement of helix 12 [9]. Rat androgen receptor was used instead of human androgen receptor, as the latter was not available in complex with the natural hormone. However, in the ligand binding region, the human and the rat protein have identical sequence and the two structures superpose with a RMSD of 0.7Å.

The steroid hormone binding pocket is a hydrophobic buried cavity. Visual inspection and structure superposition of the three receptors showed that the overall geometry of the binding pocket is conserved. A preponderance of hydrophobic residues can be observed. Hydrophilic or charged residues stabilize the ligand interaction through hydrogen bonds with the hydroxyl group of steroid molecules.

**Figure 1:**
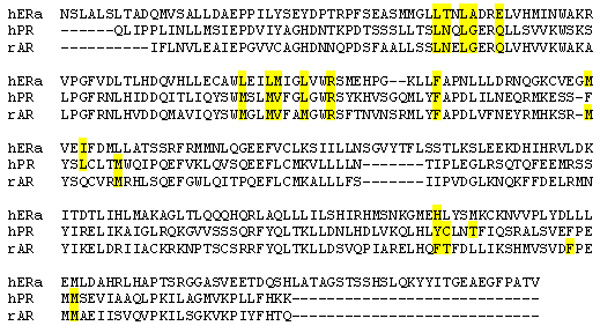
**Multiple alignment of the ligand binding domains of (hERα), (hPR) and (rAR)**. Residues involved in ligand interactions are highlighted in yellow.

Multiple alignment of hERα, hPR and rAR LDBs sequences is shown in figure 1. The overall sequence identity of (hERα) is 29% with (hPR) and 25% with (rAR), while (hPR) and (rAR) have 55% of identical residues. Aminoacid residues involved in ligand interaction, as deduced from the analysis of crystallographic structures, are highlighted in yellow. The positions involved in the binding are almost corresponding in the alignment of the three structures.

**Figure 2:**
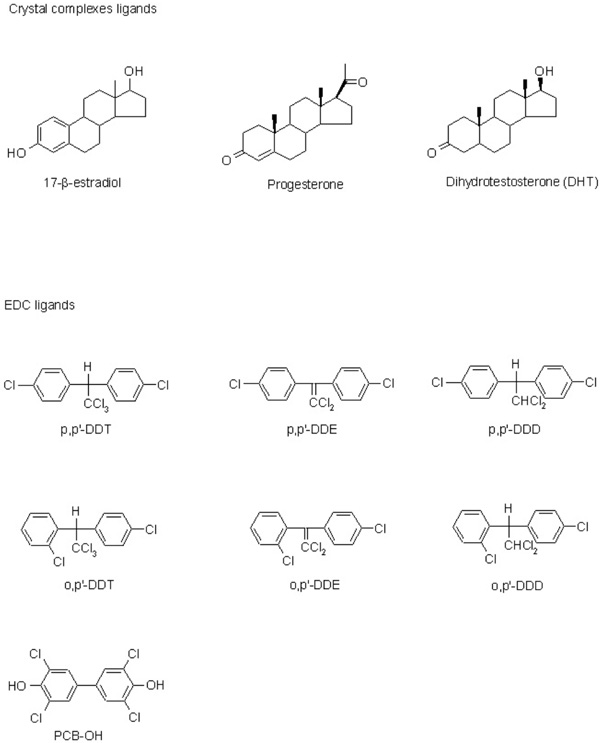
Molecular formulas of ligand compounds.

Natural hormone ligands and a set of seven EDC molecules (figure 2), were docked into the hormone binding pocket of hERα, hPR and rAR with the purpose of studying the interaction at a molecular level.

### Docking simulations

We have used AutoDock3.05/ADT [10,11] and QXP [12] programs to investigate the binding of ligands to receptors. A preliminary global docking of the hormone ligands, obtained from the crystal structures of each receptor, was performed with AutoDock using a grid encompassing the whole protein surface. For each receptor we ran a docking experiment consisting of 100 simulations, which were ranked in order of increasing docking energies values and grouped in clusters of similar conformation (RMSD 0.5 Å). For all hormone ligands, the lowest energy solutions, superposed to the crystallographic structures, displayed RMDS values from 0.49 to 0.6 Å, thus confirming the agreement of the computational procedure applied with X-ray experiment results. For the EDC ligands, the hormone binding site was investigated with a local docking procedure using a smaller grid, focussed on the binding region. As a control, the X-ray ligands were also re-run in local docking.

In a few cases (17-β-estradiol, Progesterone, Dihydrotestosterone, p,p-DDE in hERα and PCB-OH in hPR), a single cluster of solutions was found, among which the possible binding conformation within active site will be probably present.

In most cases, the solutions were grouped by AutoDock in several clusters (3–10) with comparable binding energies. From the structural analysis of the best solutions (lowest energy) of each cluster, we could highlight differences in the binding orientation.

Results from AutoDock simulations did not support the hypothesis of a single binding mode of most EDC ligands in complex with steroid receptors.

The AutoDock results were subjected to refinement with the QXP program that allows the simultaneous flexibility of ligand and active site chains. In addition, QXP allows for the evaluation of the different energetic contributions included in the energy function, giving the possibility of a more specific comparison between different solutions.

**Figure 3:**
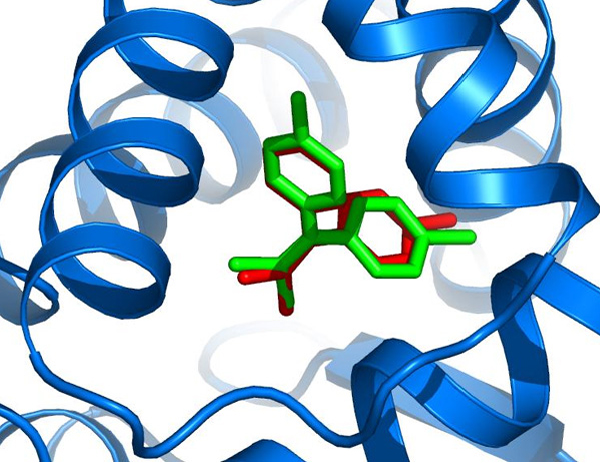
**Comparison of AutoDock (red) and QXP (green) ligand conformation prediction for p,p-DDE docked to hERα**. The solutions superposed with a RMSD = 0.015 Å.

QXP always confirmed AutoDock conformation prediction when a unique solution was found. An example is shown in figure 3 where the results of the two methods are compared in the case of p,p-DDE docked into hERα.

**Figure 4:**
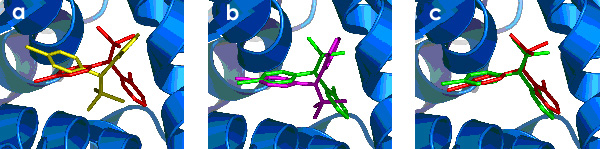
**Comparison of ligand conformation prediction for o,p-DDT docked to hERα. **Panel a. Best scored solutions with AutoDock. Calculated binding energies were -31.1 kJ/mol for solution 1 (red) and -30.8 kJ/mol for solution 2 (yellow). Panel b. Corresponding refinement with QXP: assigned total binding energies were -22.2 kJ/mol for pose 1 (green) and -15.5 kJ/mol for pose 2 (violet). Panel c. Structural superposition of pose 1 from AutoDock and pose 1 from QXP (RMSD = 0.087 Å).

When multiple conformations were obtained with AutoDock simulation, QXP was able to predict, in most but not all cases, a single binding mode. In these cases, the lowest energy conformation was accepted. Figure 4 reports the example of docking simulation of o,p-DDT docked to hERα. AutoDock results consisted of two solutions with equivalent energy values (-31.1 vs. -30.8 kJ/mol) but different binding mode (figure 4a). QXP refinement of the two AutoDock solutions allowed to define a unique binding mode by assigning different energy values (-22.2 vs. -15.5 kJ/mol) to the two complexes (figure 4b). The binding conformation associated to the lowest QXP energy prediction is the selected solution. Its binding mode superposed to an AutoDock result with a RMSD of 0.087 Å (figure 4c).

In few cases, (namely: o,p-isomers for hPR and p,p DDT, p,p DDD for hAr), QXP energy evaluation was not sufficient to discriminate between different solutions. As for these ligands no structural or mutagenesis data are available, we could not rely on experimental indications to select the most likely binding mode. Therefore, an evaluation of hydrophobic contributions as long as visual inspection and comparison of complexes to find a similarity of orientation within an isomers group, were also used as choice criteria. The final docking results used to analyze the binding mode of ECD inside hormone receptors satisfied the described criteria.

### Binding mode

**Figure 5:**
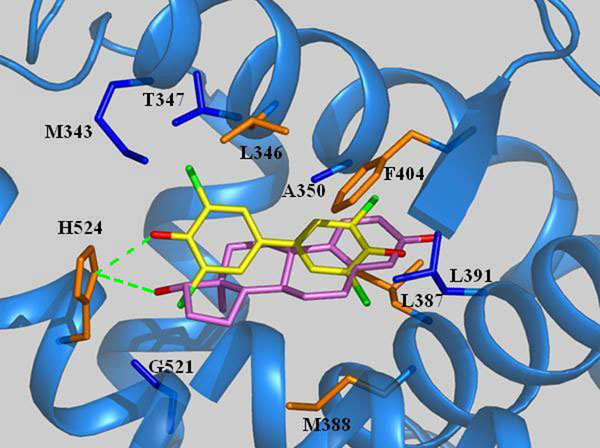
**Predicted binding of PCB-OH with hERα**. Yellow: PCB-OH, Violet : estradiol; ligand oxygen atoms are in red, and chlorine atoms in green. Aminoacids color code: orange: residues involved in ligand binding common to estradiol and PCB-OH; blue: PCB-OH specific interaction.Green dashed lines: hydrogen bonds

To study the mode of binding of EDC ligands, the best docked complexes were subjected to LIGPLOT [13] analysis that allows to identify and represent the ligand-protein contacts. Residues involved in ligand binding are reported in table 1. As expected, the type of interaction is mainly hydrophobic, due to the lipophilic character of the ligands. Hydrophilic residues are involved in the binding of PBC-OH that establish a H-bond through a hydroxyl group. As an example, the complex with hERα is represented in figure 5. Estradiol is superposed for comparison. Some of the residues involved in ligand binding are common to estradiol (orange in figure 5), including H524, which forms hydrogen bonds with both ligands.

**Figure 6:**
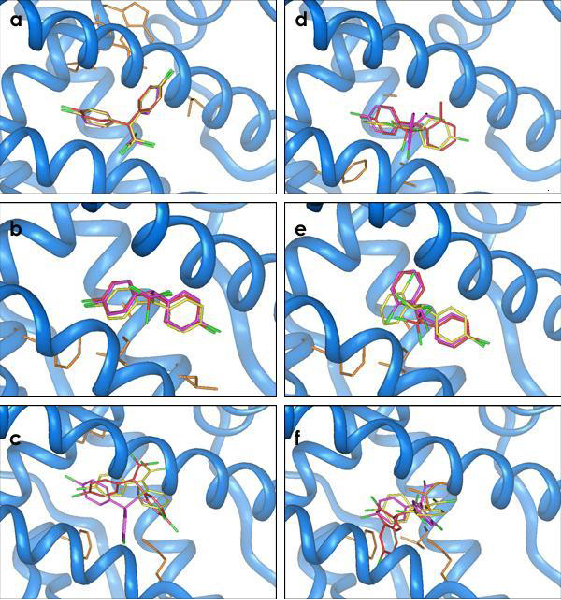
**Docking results for p,p-isomers on the left and o,p-isomers on the right**. Panels a and d refers to hERα, b and e to hPR, c and f to rAR. Residues shared by p,p- and o,p-isomers respectively for each receptor are coloured in orange. Ligands color code: red:p,p- and o,p-DDT; yellow: p,p- and o,p-DDE; purple: p,p- and o,p-DDD; green: chlorine atoms.

We could not find an overall consensus for the residues involved in EDC binding. However, the two groups of isomers were found to establish contacts with common residues within a specific receptor. p,p-DDx ligands showed a pattern of interaction with hERα involving 5 common residues and some partially shared interactions (see table 1). They displayed a very similar orientation in the binding pocket (figure 6a), and the superposition of the docked conformers gave RMSD values ranging from 0.047 to 0.074 Å. A similar result was obtained for hPR (figure 6b). In the case of binding to androgen receptor, a group of 3 common residues were involved. Ligands orientation was similar for p,p-DDT and p,p-DDE with the two phenyl rings slightly rotated, while p,p-DDD was translated with respect to the others two (figure 6c).

Predicted orientations of o,p-isomers showed similarities only when bound to progesterone receptor: corresponding portions of the ligand molecules were involved in the contacts with protein (figure 6e). Binding to estrogen and androgen receptors was predicted with markedly different orientations of ligands that shared contacts with common residues but involving different portions of the molecule (figure 6d and 6f). This behaviour is in contrast with the hormone molecule which is rigid and almost planar while DDx are flexible and can assume several possible orientations. Moreover they lack of the presence of those hydroxyl groups that anchor the hormone in the binding site through hydrogen bonds with charged or hydrophilic groups. Finally, the EDC ligands, highly hydrophobics and smaller than hormones, can be accommodated with multiple conformations in a wide binding cavity mainly lined by hydrophobic residues. Because of these characteristics, the specificity of binding is lower than for hormone ligands. We found that QXP calculated free energies of binding were in all cases higher for EDC than for hormone ligands (table 2): for example in hERα, binding energy raised from -36.7 to -13.4 kJ/mol from estradiol to p,p-DDT. The only exception is the o,p-DDE bound to hPR for which a ΔG value comparable to progesterone was found (-25.0 vs. -26.3 kJ/mol). In general, o,p-isomers show higher affinity for all tested receptors compared to p,p-DDx and PCB-OH. Our data confirm the hypothesis of mild toxicity of EDC, in agreement with evidences from competition experiments [14,15]. However, because of the low selectivity and great adaptability, EDC toxicity might be amplified by a synergic effect.

## Conclusion

The purpose of this work was to describe the molecular interactions between steroid receptors and some endocrine disrupting pollutants. The complexes were modelled starting from the X-ray coordinates of the receptors and simulating the ligand binding by a combination of flexible docking. Subsequent structural analysis of the models provided information on the binding mode. In general, this was characterized by multiple hydrophobic contacts which engaged a different number of residues facing the binding pocket, depending on the ligands orientation. The EDC ligands did not display a unique mode of binding, probably due to their lipophilicity, their flexibility and their small volume, which conferred them a great adaptability in the hydrophobic and large binding pocket of steroid receptors.

A wider exploration of the binding of steroid receptors with other classes of EDC compounds is already in progress. Further investigations of their binding properties at molecular level will provide useful information for the prediction of the toxicity of compounds that are released in the environment and also for the rational design and synthesis of new molecules with low impact on human health.

## Methods

### Preparation of the ligand and receptor molecules for docking

Molecular structures of the EDC ligands (namely p,p- DDT, o,p-DDT, p,p- DDE, o,p-DDE, p,p- DDD, o,p-DDD, PCB-OH) were built and energy minimized with MacroModel [Schrödinger LLC and 16 ], the resulting geometries were re-optimized with semi-empirical quantum mechanic calculations, using the Hamiltonian AM1 as implemented in Spartan 'O2, (Wavefunction Inc., Irvine, CA).

To model the receptor-ligand complexes, the coordinates of human estrogen α (hERα) [PDB:1G50], progesterone (hPR) [PDB:1A28] and rat androgen (rAR) [PDB:1I37] receptors in complex with the corresponding steroid hormone (estradiol, progesterone, and dihydrotestosterone respectively) were used.

The receptors and the ligand were prepared for docking using ADT, the AutoDock tool graphical interface [11]. For each receptor structure polar hydrogens were added, Kollman charges were assigned and atomic solvatation parameters were added. For the ligands, polar hydrogen charges of the Gasteiger-type were assigned and the non-polar hydrogens were merged with the carbons. The internal degrees of freedom and torsions were set for each EDC.

### Docking simulations

A two-step docking protocol was employed. In a first phase, each inhibitor was docked into the active site by means of the program AutoDock3.05 [10] with the macromolecule held fixed and the ligands being flexible. The region of interest used by AutoDock was first the whole receptor protein and then was defined in such a way to include a specific portion of the binding site of the macromolecule, as described above. A smaller grid, focussed on the binding region, was used and the number of simulations was set to 50. In particular, affinity maps for all the atom types present, as well as an electrostatic map, were computed with a grid spacing of 0.375 Å. The search was carried out with the Lamarckian Genetic Algorithm: populations of 250 individuals with a mutation rate of 0.02 have been evolved for 10 millions generations. Evaluation of the results was done by sorting the different complexes with respect to the predicted binding energy. A cluster analysis based on root mean square deviation values, with reference to the starting geometry, was subsequently performed.

This first step approach served only to accommodate the different ligands into the binding site. The structural models collected from the lowest-energy docking solution of each cluster of AutoDock, have been used as input for QXP docking [9]. The algorithm implemented in the QXP program allows for fully flexibility of the inhibitors and simultaneous flexibility of the active site side-chains. The starting structure was previously optimized by energy minimization. Then, each docking run included 50 cycles of Monte Carlo perturbation, subsequent fast searching, and final energy minimisation. For each single docking QXP simulation the results were evaluated in terms of total estimated binding energy, internal strain energy of the ligand, van der Waals and electrostatic interaction energies.

All calculations were performed on Octane and O2 SGI workstations and on Linux Cluster of 9 workstations each equipped with 2 processors AMDx64 and 2 GB RAM.

Figures were realized with InsightII (Accelrys) and PyMOL Molecular Graphics System (DeLano Scientific, San Carlos, CA, USA).

## Authors' contributions

PD performed docking simulation, supervised data analysis and drafted the manuscript. ES participated to docking analysis, identification of receptor-ligand interaction and prepared all figures. PF contributed to data analysis and manuscript preparation. LM contributed to manuscript preparation and revision. ER conceived the project, coordinated its execution and prepared the manuscript. All authors read and approved the final manuscript.

## Figures and Tables

**Table 1:** Protein-ligand interactions obtained by LIGPLOT analysis of X-ray and QXP docked complexes. Bold and underlined: Residues shared by p,p- and o,p-isomers respectively for each receptor.

	hERa	hPR	rAR
Estradiol	L346,E353,L387,M388, R394, F404,M421,H524L525,L539		

Progesterone		L718,N719,Q725,M759, R766,L797,M801,Y890, C891,T894,M909	

Testosterone			L704,N705,L707,G708, Q711,M742,M745,M749, R752,F764,M780,M787, F876,T877, F891,M895

p,p-DDT	**W383,L384,L387,G521,L525,** A350,L391	**L715,L718,F778 ** N719,G722,F905	**M745,F764,M780 ** L704,L707,W741

p,p-DDE	**W383,L384,L387,G521, L525 ** F404	**L715,L718,F778 M756**	**M745,F764,M780 ** W741, M742, L873, T877

p,p-DDD	**W383,L384,L387 G521, L525 ** L346, A350, F404,L540	**L715,L718,F778 ** C891, F905, M909	**M745, F764,M780 ** L704, L707, M787,L873

o,p-DDT	L346,A350, F404,M421 M343,L349,L384,L387,M388, I424,F425,L428,G521,H524, L525	L718,F778 L715,N719,G722,W755, M756,M759,F794,M801 T894,V903,F905,M909	L704, F764,M780,L873 L707, M787

o,p-DDE	L346,A350,F404,M421, M343,L525	L718, F778 T890, C891, T894, F905	L704, F764,M780,L873 W741, M745, F876, T877

o,p-DDD	L346,A350, F404,M421 L384, L387, M388, I424, F425, L428	L718, F778 L721, M759, F794, L797, Y890, M909	L704, F764,M780,L873 L707,M742, M745,F876

PCB-OH	M343,L346,T347,A350 L387, M388,L391,F404 G521,H524	L715,L718,L721,V760, L763,F778, L797,M801 Y890,C891	L701,L704,N705,L707, Q711,W741,M742 M745, F764,L873,F876

**Table 2:** Estimated binding energies (kJ/mol) obtained by QXP simulation for tested ligands docked to steroid receptors.

	hERα	hPR	rAR
1) Estradiol	-36.7		
2) Progesterone		-26.3	
3) Testosterone			-30.4
4) p,p-DDT	-13.4	-16.6	-14.8
5) p,p-DDE	-10.7	-14	-13.4
6) p,p-DDD	-12.4	-17.1	-12.7
7) o,p-DDT	-22.2	-17.8	-22.8
7) o,p-DDE	-19.2	-25.0	-12.8
9) o,p-DDD	-17.3	-14.0	-15.9
10) PCB-OH	-8.6	-10.6	-14.7
